# Immune discrimination between commensals and pathogenic bacteria

**DOI:** 10.1080/21505594.2026.2677297

**Published:** 2026-05-22

**Authors:** Hamad H. Alanazi

**Affiliations:** Department of Clinical Laboratory Sciences, College of Applied Medical Sciences, Jouf University, Al-Qurayyat, Saudi Arabia

**Keywords:** Microbiota, immune response, pathogenic bacteria, commensal bacteria, gut microbiome, host–microbe interactions

## Abstract

Interaction with various bacteria is essential for the development of various components of the immune system. To prevent disease, the immune system must continuously discriminate between commensal and pathogenic bacteria. The immune system employs several mechanisms to discriminate between beneficial and harmful bacteria. This ensures selective immune tolerance toward commensals, especially in the gastrointestinal tract. Both commensal and pathogenic bacteria contain features that provoke immune responses. However, how the immune system reacts to or eliminates bacterial infections while preserving commensals is not fully understood. This review aims to explore the underlying mechanisms used by the immune system to distinguish between commensals and pathogenic bacteria. The review also addresses how commensals interact with immune system components to facilitate immune discrimination and host protection. Finally, dysbiosis and therapeutic interventions used to restore microbial balance are also discussed in this review.

## Introduction

### Immune recognition of bacteria

The immune system is highly equipped with diverse range of receptors to fight infectious agents, including bacteria, viruses, and fungi. Bacteria can be divided into commensal or pathogenic bacteria. Each site of the body is vulnerable to bacterial infections and therefore has some form of immunological barrier. The skin is an immunological physical barrier that protects the body from bacterial infections. Mucus membranes, gastric acidity, and other chemical barriers prevent pathogenic bacterial entry. Once bacteria breach immunological barriers, they face different forms of immunity, mainly neutrophils or macrophages [1]. Neutrophils play crucial roles in engulfing microbes, especially bacteria, and destroying them via reactive oxygen species (ROS) and other degrading molecules [2]. Neutrophils are the first responders to bacterial infections, and their increase in the blood is usually a sign of bacterial infections in patients [3]. Once bacteria invade the host after bypassing physical barriers, their components will be detected by several immune receptors. Epithelial cells and resident immune cells express Toll-like receptors (TLRs) or nucleotide-binding oligomerization domain-like receptors (NLRs) [4]. The primary function of these receptors is to recognize pathogen-associated molecular patterns (PAMPs) [5]. Once phagocytic cells detect microbes through their pattern recognition receptors (PRRs), they engulf and phagocytose microbial particles, producing alarming cytokines and other molecules that enhance the inflammatory process and recruit leukocytes [6]. In addition, complement proteins contribute to the inflammatory process and the elimination of bacteria by lysing bacterial cell membranes [7]. Subsequently, when innate immune responses fail to clear the bacterial agent, adaptive immunity takes over [8]. Dendritic cells and macrophages take up antigens and migrate to the draining lymph nodes, where they present the antigens to CD4+ T lymphocytes (T cells) [9,10]. T cells have specific receptors called T-cell receptors (TCRs). The immune system generates numerous TCRs, each with specificity for one antigen [11]. Once that antigen is encountered, T cell proliferates into several clones to fight the bacterial agent [12]. On the other hand, B lymphocytes (B cells) also recognize bacterial antigens through their B-cell receptor (BCR) [13]. Upon encountering bacteria, B cell proliferates and differentiate into plasma cell, which produce antibodies [14]. Antibodies play a significant role in defense against bacterial infections [15]. They prevent bacterial replication, bacterial attachment to host cells, and contribute to neutralization of their toxins [16]. After the bacterial agent is eliminated, a few memory T and B cells remain to mount future responses against the same microbe [17]. Immune recognition by innate and adaptive immune cells contributes to discrimination between commensals and pathogenic bacteria. This review aims to explore the latest updates in our understanding of how the immune system discriminates between commensals and pathogenic bacteria and how commensals interact with immune system components to provide protection from disease.

### Discrimination between commensals and pathogenic bacteria

**Figure 1.**
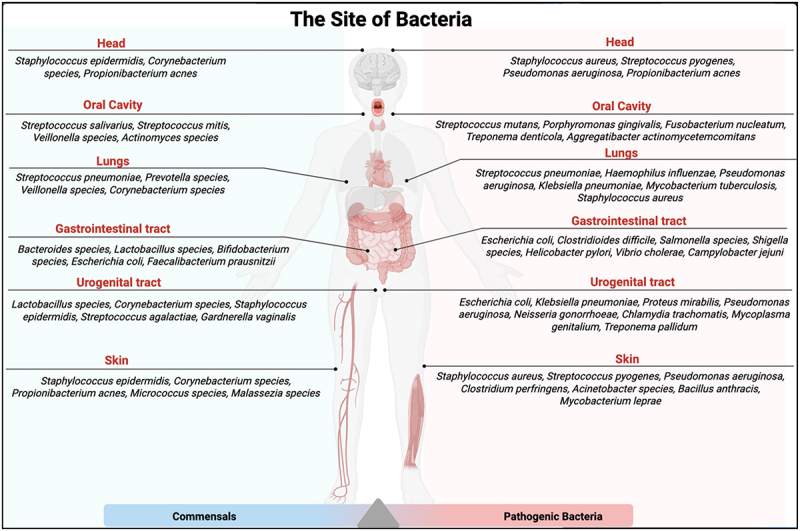
Microbial composition across human body organs. The figure illustrates the distribution of commensals and pathogenic microorganisms across various organs in the body, including the head, oral cavity, lungs, gastrointestinal tract, urogenital tract, and skin. The head, urogenital tract, and skin microbiota harbor commensals such as *staphylococcus epidermidis*, among others. *E. coli* is a common pathogenic bacterium found in the gastrointestinal and urogenital tracts. The equilibrium between these beneficial bacteria and opportunistic pathogens is important for maintaining body homeostasis and preventing bacterial infections.

The immune system responds to antigens from both harmless and harmful bacteria; however, the outcomes differ depending on the nature of the organism. Commensals typically trigger slight immune responses essential for maintaining immune tolerance, while pathogenic bacteria trigger stronger immune responses that lead to inflammation and robust immune activation. Trillions of commensals exist in different places in the body, including the head, gut, skin, oral cavity, respiratory tract, and the urogenital tract [18,19]. The majority of commensals are found in the colon of the gut [20]. As shown in Figure 1, overall health and homeostasis can be maintained in the presence of both commensals and pathogenic bacteria under balanced conditions. The innate immune system tolerates commensals in many ways. For example, physical barriers, such as the epithelial layer and mucus, separate commensals from invading host tissues. Table 1 shows how the immune system relies on different mechanisms to discriminate between commensals and pathogenic bacteria, ensuring immune tolerance to commensals and protection against pathogenic bacteria.

**Table 1.** Examples of immune discrimination mechanisms between commensals and pathogenic bacteria.

Mechanism	Commensal bacteria	Pathogenic bacteria	References
Separation	Commensals are usually confined in a space like the gut lumen or the mucosal layer	Pathogens invade or breach barriers like the gut lumen or the mucosal layer	[21]
Toxin production	Commensals usually do not produce harmful toxins, but instead they produce bacteriocins (e.g. certain *lactobacillus* strains) that inhibit pathogens like *Listeria*	Pathogenic bacterial strains like *Shigella dysenteriae*, *Vibrio cholerae*, and *Helicobacter pylori* produce harmful toxins such as Shiga toxin, cholera toxin, and VacA toxins, which cause disease by damaging the host tissues	[22,23]
Cytokines	Commensals stimulate the production of low levels of anti-inflammatory cytokines (e.g. IL-10, TGF-β)	Pathogenic bacteria usually trigger the production of high levels of pro-inflammatory cytokines (e.g. IL-1β, TNF-α, IL-6)	[24,25]
Activation of dendritic cells	When dendritic cells take up commensals, they promote immune tolerance by inducing Tregs and anti-inflammatory cytokines	When dendritic cells take up pathogenic bacteria, they mature and present co-stimulatory molecules to activate T cells, which leads to inflammation and pathogen elimination	[26–29]
Antibodies	Commensals typically induce IgA production, which neutralizes them and keeps them at the mucosal layers	Pathogenic bacteria induce an IgG immune response, which promotes inflammation and the elimination of pathogens	[30,31]

PRRs, such as TLRs and NLRs, respond to commensal components and stimulate tolerance rather than inflammatory responses, which typically occur when encountering pathogenic bacteria [32,33]. In addition, innate immune cells like macrophages and dendritic cells contribute to immune regulation and tolerance through the production of anti-inflammatory cytokines such as IL-10 when responding to commensals. On the other hand, adaptive immunity responds to commensals in some ways, such as the production of secretory IgA in mucosal tissues, which binds to and inhibits commensals from invading epithelial cells. Moreover, many commensals stimulate regulatory T cells (Tregs) to prevent inflammation and maintain immune regulation [34].

### Bacterial strategies to avoid immune discrimination

The immune system’s primary goal is to protect against all invading pathogens through innate (e.g. skin, mucus, cytokines, antimicrobial peptides) and adaptive immune components (e.g. lymphocytes). Bacteria cause several diseases, including strep throat infections, urinary tract infections (UTIs), skin infections, otitis media, sinusitis, cholera, tuberculosis, chlamydia, and gonorrhea. To escape immune discrimination, bacteria have developed various strategies to overcome the immune system. For example, *Mycobacterium tuberculosis* use the host cells as a shield to evade immune defenses [35]. Many other bacterial organisms avoid recognition by the immune cells through producing capsules to avoid phagocytosis, or proteins like IgA protease to cleave IgA and inhibit its function. Furthermore, pathogenic bacteria rely on several types of proteins, including hyaluronidases, proteases, streptolysins, protein M, catalase, collagenase, coagulase, hemolysins, lecithinase, and exotoxins, to help them survive and evade the immune system. In addition, bacteria can overcome the complement system, a key element in inflammation and immune defense against pathogenic bacteria.

Contrary to pathogenic bacteria, commensals produce molecules that facilitate resistance to bacterial infections and maintain microbial balance. For instance, *Bacteroides fragilis* in the colon stimulates regulatory T cells, which help maintain balance and reduce inflammatory response [36]. Moreover, *Staphylococcus epidermidis* residing on the skin produces antimicrobial peptides (AMPs) that kill pathogens like *Staphylococcus aureus.*

### The role of microbiota in facilitating immune discrimination and host defense

Microbiota play a vital role in host defense and immune system development through several mechanisms. Commensal microbiota found on the skin or lining the gut prevent the growth of pathogenic bacteria by competing for space and nutrients and producing substances that inhibit microbial growth, such as bacteriocins, AMPs, and short-chain fatty acids (SCFAs). In addition to their role in defense against pathogens, they play a role in homeostasis by educating and maturing the components of the immune system. The exposure of immune system components to commensals helps maintain immune tolerance by inducing the activation of Tregs to produce anti-inflammatory cytokines such as IL-10 and TGF-β, thus preventing the development of chronic inflammation or autoimmune diseases.

**Figure 2.**
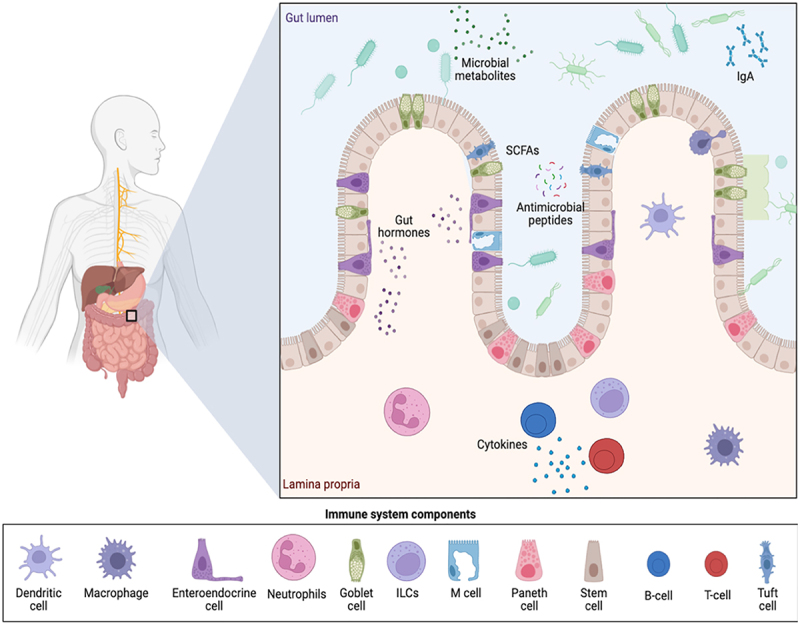
Immune and microbial interplay in the intestinal lamina propria and gut lumen. The illustration shows the anatomical and immunological components of the intestinal lamina propria and the gut lumen. Microbial constituents, including commensals, and microbial metabolites interact with epithelial cells and mucus layers to shape immune responses. Subsequently, immune cells such as dendritic cells, macrophages, innate lymphoid cells, B cells, and T cells within the lamina propria signal for the production of important cytokines and antimicrobial peptides. Host and commensal crosstalk ensures gut protection through balanced immune responses.

The microbiota influences the development of immune cells in several ways, especially in the gut. They help differentiate and mature naive T cells into various subsets, such as T helper 17 (Th17) or Treg cells, which are essential for maintaining gut homeostasis [37,38]. In gut-associated lymphoid tissue (GALT), specialized cells known as microfold cells (M cells) capture foreign pathogens and transport them across the epithelial barrier from the gut lumen. Dendritic cells also extend to the gut lumen and capture and engulf pathogens. Dendritic cells migrate to Peyer’s patches or mesenteric lymph nodes, thus allowing for the production of inflammatory cytokines and initiation of adaptive immune responses mediated by T and B cells. Based on the nature of the pathogen, T cells differentiate into helper T cells (CD4+) and cytotoxic T cells (CD8+). The former assists in activating B cells against extracellular bacteria, while the latter specializes in defending against intracellular bacteria. B cells differentiate into plasma cells, the producers of immunoglobulins (IgGs). The main Ig produced by B cells in the gut during infection with extracellular bacteria is IgA because of its essential function in neutralizing bacterial toxins on mucosal surfaces [39]. Commensal bacteria can also be transported from the lumen through M cells. Dendritic cells process these antigens and present them to T cells, which helps maintain immune tolerance. Studies have shown that mice lacking M cells exhibit microbiota dysbiosis [40]. Additionally, other immune cells that interact with microbiota, like goblet and tuft cells, have an essential role in maintaining immune tolerance in the gut. Goblet cells produce mucins (e.g. MUC2), which form the mucus layer that separates the microbiota from epithelial cells [41,42]. In addition, goblet cells transport luminal antigens into antigen-presenting cells [42]. Moreover, tuft cells respond to helminths and protozoa by secreting IL-25, which recruits and activates ILC2s and type-2 T helper cells [43]. Tuft cells were recently shown to have a role in eradicating *Shigella* bacteria by sensing bacterial metabolite N-undecanoylglycine (N-C11-G) [43]. The intestine is the largest immune organ where microbes and immune cells interact [44]. Figure 2 shows the immune and microbial interplay in the intestinal lamina propria and gut lumen. The interaction between commensals and immune cells leads to activation of immune responses that are beneficial to the host.

### Immune discrimination in the gut

**Figure 3.**
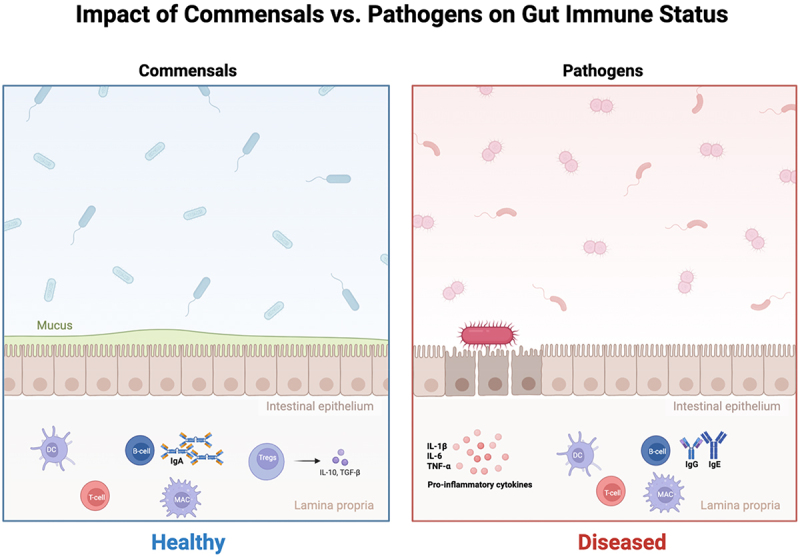
B and T cell-mediated immune responses to commensal and pathogenic bacteria in the gut. Illustration of the immune system’s response to commensal versus pathogenic bacteria within the intestinal environment, highlighting key immune cell types, cytokines, and barrier dynamics. The left side shows healthy intestinal homeostasis and tolerance, where commensals induce Tregs, IgA secretion, and maintenance of epithelial integrity through production of anti-inflammatory cytokines such as IL-10 and TGF-β. In contrast, the right side shows diseased state and inflammatory responses induced by pathogenic bacteria, including the secretion of IgG, IgE, and pro-inflammatory cytokines (e.g. TNF-α, IL-6). The type of microbe determines the immune status of the gut.

The immune system in the gut reacts differently to pathogenic and commensal bacteria. PRRs play a vital role in mounting appropriate immune responses through immune cells such as macrophages and dendritic cells [45]. PAMPs found on commensal bacteria are recognized by numerous PRRs [46]. This recognition triggers the initiation of a signaling cascade, such as NF-kB and MAPK, gene expression, and cytokine production [46]. One of the crucial PRRs is the family of TLRs. TLRs recognize bacterial components; for example, TLR2 recognizes lipoproteins, lipoteichoic acid (LTA), and peptidoglycans (PGs). TLR4, TLR5, and TLR9 recognize lipopolysaccharides (LPS), bacterial flagellin, and unmethylated CpG DNA, respectively [47]. The recognition and binding of the receptors to their appropriate ligands results in the activation of downstream transcription factors and the expression of pro-inflammatory or anti-inflammatory cytokines. TLR receptors can be activated by their ligands found in both commensal and pathogenic bacteria [48]. Gastrointestinal homeostasis is thought to be achieved through the sequestration of commensals by the epithelial layer [49]. The mechanisms by which the immune system in the gut is not activated to a level where it produces immune responses against commensals are not fully understood. Activation of Tregs by commensal antigens minimizes inflammation in the stomach through the production of IL-10 and TGF-β [50]. Figure 3 depicts how commensals promote intestinal homeostasis and immune tolerance through inducing Tregs and IgA production, in contrast, to pathogenic bacteria which induce inflammatory responses characterized by increased pro-inflammatory cytokines.

Although the immune system is minimally activated, immune responses are regulated and contribute to homeostasis, especially in the gastrointestinal tract. Dysregulation of homeostasis and chronic inflammation in the gut has been linked to alterations in commensal bacteria. In addition, commensals contribute to the integrity of the immune system by activating goblet cells to produce mucus and AMPs.

Several pathogenic gram-positive and gram-negative bacteria, such as *Staphylococcus aureus*, *Salmonella enterica*, *Escherichia coli*, and *Clostridium difficile*, cause skin infections, gastroenteritis, UTIs, and antibiotic-associated diarrhea, respectively. Pathogens are usually equipped with virulence factors such as proteases, adhesins, pili, biofilms, hemolysins, LPS, and capsules, which facilitate their penetration of the epithelial layer and activation of several immune sensors, such as TLRs, RIG-I, and NOD-like receptors [49,51]. Usually, bacterial invasion is prevented by the skin or mucus; however, when bacteria escape physical barriers, they are phagocytosed by innate cells like neutrophils, which kill bacteria by releasing ROS. Moreover, complement proteins also contribute to bacterial killing by opsonizing bacteria to facilitate their destruction or the formation of a membrane attack complex (MAC), which results in the leakage of bacterial cell contents. When bacterial replication persists, adaptive immune cells, such as B cells, increase bacterial elimination by enhancing antibody production and activating macrophages.

The immune system responds to bacteria based on their specific virulence factors. For example, adhesins, pili, and fimbriae, which enable bacterial attachment, are prevented by mucus and secretory IgA. In addition, the complement protein C3b opsonizes the bacterial capsules to facilitate bacterial clearance through phagocytosis by macrophages. Additionally, antibodies such as IgA or IgG neutralize bacterial toxins such as *diphtheria* or *cholera* to prevent them from causing tissue damage. Some bacteria, such as *Helicobacter pylori*, have flagella, which promote motility and invasion. The immune component responsible for defense against flagella is TLR5, which triggers the activation of innate immune responses.

Commensal bacteria activate a wide range of immune receptors, which result in the initiation of signaling pathways that lead to cytokine production or immune cell recruitment. For instance, *Bacteroides fragilis* stimulates the production of interferons (IFNβ) through activating NOD2 [52]. Additionally, *Lactobacillus* can enhance the recruitment of dendritic cells and release of immunoregulatory cytokines (IL-10 and TGF-β) by engaging TLR9 [53]. Moreover, commensal-derived metabolites (e.g. taurine, histamine, and spermine), produced by *Bacteroides* and *Lactobacillus* species, induce production of TNF-α, IL-18, and AMPs via the NOD-like receptor family pyrin domain containing 6 (NLRP6) [54,55].

### Microbiota-mediated pathogen inhibition

The human microbiota comprises approximately 100 trillion microbial cells, most of which reside in the gut [56]. Majority of gut microbiota belong to two phyla: *Firmicutes* and *Bacteroidetes* [57]. The gut microbiota offers host protection by colonization resistance via competitive exclusion [58]. The more healthy bacteria lining the gut, the fewer opportunities for harmful bacteria to cause disease. Several commensals, such as *Lactobacillus* spp. and *Bifidobacterium* spp. inhibit pathogenic bacteria (e.g. *E. coli*, *Salmonella*), as shown in Tables 2 and 3.

**Table 2.** Examples of commensals that inhibit pathogenic bacteria.

Commensal Bacteria	Pathogenic Bacteria Inhibited	References
*Lacticaseibacillus rhamnosus* GG	*Salmonella*	[59]
*Lactobacillus rhamnosus* SQ511	*Salmonella enteritidis*	[60]
*Bifidobacterium longum* K5	Enterohaemorrhagic *Escherichia coli* O157:H7	[61]
*Lactiplantibacillus plantarum* TW57-4	*Listeria monocytogenes*	[62]
*Corynebacterium accolens*	*Streptococcus pneumoniae*	[63]
*Corynebacterium accolens*	*Staphylococcus aureus*	[64]
*Clostridium scindens*	*Clostridioides difficile*	[65]
*Urinary lactobacilli*	Uropathogens: *Escherichia coli* (*E. coli*), *Klebsiella pneumoniae* (*K. pneumoniae*), *Enterococcus faecalis* (*E. faecalis*)	[66]

**Table 3.** Examples of microbiota-mediated pathogen inhibition.

Commensals	Inhibition mechanisms	Location	Reference
*Lactobacillus* species, including *Lacticaseibacillus paracasei* and *Lactiplantibacillus plantarum*	*Lactobacillus* prevents the growth of *Streptococcus mutans* (*S. mutans*), which is the primary pathogen responsible for dental caries and decay.	Oral cavity	[67]
*Staphylococcus lugdunensis*	*Staphylococcus lugdunensis* produces lugdunin, which limits skin and nasal colonization by *Staphylococcus aureus.*	Nasal cavity	[68,69]
*Enterococcus faecalis*	*Enterococcus faecalis* strain inhibits *vancomycin-resistant Enterococcus* (VRE) through bacteriocin.	Intestinal tract	[69,70]
*Bacteroides fragilis, Lactobacillus species, Faecalibacterium prausnitzii*	Many bacteria stimulate mucus production through the activation of goblet cells.	Gut	[71–74]
*Akkermansia muciniphila*	*Akkermansia muciniphila* stimulates mucus production, enhances the mucosal barrier to prevent pathogen entry, strengthens tight junctions, and promotes the expansion of Tregs.	Gut	[75–77]
*Bacteroides fragilis, Bifidobacterium species, Lactobacillus species,*	Interaction of commensal bacterial antigens with B cells results in the stimulation of B cells to differentiate into plasma cells.	Gut	[74,78–81]
*Lactic acid bacteria (e.g. Lactobacillus)*	Extracellular vesicles (EVs) isolated from lactic acid bacteria (e.g. *Lactobacillus*) inhibit pathogenic bacteria like *Acinetobacter baumannii*.	Gut	[82]
*Bifidobacterium species, Bacteroides species (e.g. Bacteroides thetaiotaomicron), Prevotella species* *Certain Clostridium species*	Many commensals like *Bifidobacteria* and *Bacteroides* species produce fatty acids like acetate and butyrate, which lower the pH and make the environment unfavorable for the growth of pathogenic bacteria.	Colon	[83–85]
Gut commensal microbiota	Fecal microbiota transplantation (FMT) from healthy mice decreases the burden of *L. monocytogenes* in the liver and spleen and reduces mortality.	Gut, liver, spleen	[86]

Commensal bacteria in the gut consume incoming sugars, iron, and amino acids, which limits the availability of resources to pathogenic bacteria [87]. Compared to pathogenic bacteria, commensal bacteria are highly efficient at metabolizing dietary fibers [88]. The commensal microbiota also competes for binding sites in the mucosal layer of the gut, thereby limiting the attachment of pathogenic bacteria. Once established, commensal bacteria such as *lactobacilli* produce lactic acid, creating an acidic environment, unfavorable for many pathogenic microbes like *E.coli* [89,90]. Moreover, commensal bacteria produce bacteriocins and lactic acid, which destroy harmful bacteria [91]. Bacteriocins like Nisin create pores in the membrane of pathogenic bacteria, which cause ion leakage and eventually cell death [92]. Several commensals target pathogenic bacteria through bacteriocins; for instance, *L*. *lactis* produces Nisin, a lanthipeptide that causes pores in the membrane of *S. aureus* and C. difficile [93]. P. acidilactici also produces Pediocin PA-1, which destroys *L. monocytogenes* through pore formation [93]. In addition, *E. coli* produces Colicin bacteriocin, which kills *Y. enterocolitica* [93]. Besides pore formation, bacteriocins destroy pathogenic bacteria by inhibiting the synthesis of peptidoglycan, RNA polymerase, and nucleic acid, as well as elongation factor-dependent reactions [93]. Also, many novel microcins have been recently discovered to be active against pathogenic bacteria like *E. coli* [94]. Commensals like *lactobacilli* produce hydrogen peroxide (H_2_O_2_), which inhibits the growth of *Neisseria gonorrhoeae* [95]. Several long-chain fatty acids derived by gut microbiota have been shown to inhibit important foodborne pathogens like *Salmonella* and *Listeria monocytogenes* [96]. Moreover, one of the ways to limit the growth of pathogenic bacteria is by sequestering iron. Commensals release iron-scavenging siderophores, which limit iron supplementation to pathogenic bacteria, thereby contributing to immune protection. For instance, nasal commensals have been shown to reduce *Staphylococcus aureus* proliferation by restricting siderophores [97].

Moreover, commensals like lactic acid bacteria (LAB) inhibit the growth of harmful bacteria through mechanisms like increasing gut acidity and weakening pathogen enzymes [98]. LAB ferment carbohydrates to produce lactic acid which creates acidic environment that impairs several pathogen enzymes like proteases [98]. Additionally, the presence of commensal bacteria in the gut is also essential for promoting tight junctions and fortifying the mucosal layer [99]. Commensal bacteria help maintain microbial diversity and balance, which protects against pathogenic bacteria. Any disruption of microbiota predisposes the body to various types of infections.

### Dysbiosis and immune dysregulation

Loss of microbiota diversity or a reduction in commensals leads to dysbiosis. Studies have shown that dysbiosis is linked to several diseases. For instance, altered microbial diversity between healthy and diseased groups, specifically, in *Roseburia*, *Faecalibacterium*, *Prevotella*, and *Enterococcus* species has been observed in patients with bloodstream infections [100]. Additionally, changes in *Bifidobacterium* and *Lactobacillus* species have also been associated with the severity of COVID-19 infection [101]. Furthermore, dysbiosis in the lung microbiome, previously considered absent, is linked to infections in the gut through the gut-lung axis [102].

Many pathogens can alter microbiota diversity leading to dysbiosis. Respiratory viral infections, for instance, with the influenza A virus, reduce gut microbiota and disrupt the mucosal layer [102]. Notably, bacterial LPS, when injected into mice, intratracheally, affected respiratory microbiota and facilitated the transfer of *Clostridium* into the intestine through the bloodstream in 24 h [102]. Dysbiosis has also been correlated with neurodegenerative diseases, including Alzheimer’s Disease (AD), Parkinson’s Disease (PD), and Multiple Sclerosis (MS) [103]. It was shown that intestinal dysbiosis increases the severity of PD through inflammation and oxidative stress [104].

Recently, dysbiosis has been linked to a number of diseases including irritable bowel syndrome (IBS), celiac disease, asthma, metabolic syndrome, obesity, liver diseases (hepatitis, cirrhosis), depression, diabetes (type 1, type 2, gestational), cancer (lung, oral, colorectal cancers), heart diseases (hypertension, atherosclerosis), inflammatory bowel diseases (ulcerative colitis, crohn’s disease), and chronic kidney disease [19,105,106]. A reduction or modulation of the microbiota, especially in the gut, compromises immune responses against harmful bacteria. The commensals contribute to a healthy intestinal barrier by blocking the entry and growth of pathogenic bacteria. The presence of commensals like *Lactobacillus* and *Bifidobacteria* protects the gut via the production of SCFAs, which increase the environment’s acidity. In addition, the presence of commensals supports the intestinal epithelial layer to prevent leakage of bacterial LPS and PGs into the circulation. Many pathogenic bacteria, such as *Salmonella typhimurium*, *enterohemorrhagic E. coli* (EHEC), and *P. aeruginosa*, are inhibited or killed in the presence of commensals [89,107–109]. Dysbiosis affects the maturation of immune cells, such as macrophages and dendritic cells, which are the main players in shaping adaptive immune responses during infections [110]. Given the critical role of microbiota in the immune system, their disruption is linked with several autoimmune diseases, including Sjögren’s syndrome (SS), systemic lupus erythematosus (SLE), rheumatoid arthritis (RA), and MS [111–113].

### Antibiotic-induced dysbiosis and therapeutic interventions

Antibiotics are necessary to overcome bacterial infections and prevent the consequences of secondary bacterial infections associated with viral infections, including respiratory infections such as *Streptococcus pneumoniae*. However, the repeated use of antibiotics negatively impacts the overall immunity, diversity, and balance of the microbiota, causing dysbiosis [114]. Antibiotic-induced dysbiosis is associated with conditions such as *Clostridium difficile* infection or rheumatoid arthritis (RA) [115,116]. Although antibiotics eliminate pathogenic bacterial infections, they destroy commensals. Antibiotic overuse enhances resistant bacteria’s growth, creating superbugs [117]. Reduced colonization by beneficial bacteria facilitates the development and thriving of pathogenic bacteria and fungi [118]. The consumption of probiotics can solve the issue of losing highly diverse microbiota and restore the normal balance of the healthy gut [119]. Fecal microbiota transplants from healthy individuals can also restore healthy microbiota [120].

FMT is a therapeutic intervention involving the transfer of fecal bacteria from healthy donors to the gastrointestinal tract of a patient with dysbiosis. The purpose of FMT is to restore a healthy microbiome in the gut, particularly after its disruption by antibiotics, as observed in cases of recurrent *Clostridioides difficile* infection (rCDI) [121]. FMT is currently approved by the Food and Drug Administration (FDA) for the prevention of rCDI [122,123]. Two drugs (Rebyota, Vowst) have been released as therapeutic options for rCDI patients [123]. Many FMT formulations are being investigated for the treatment of other medical conditions such as IBD, obesity, IBS, Type 2 diabetes, Graft-versus-host disease, and arthritis [124,125]. Although FMT has shown promising results in treating several medical conditions, there are still obstacles in implementing it as a standard therapy due to regulatory and safety concerns [125].

Specific strategies can mitigate antibiotic-induced dysbiosis, including phage therapy (PT), narrow-spectrum antibiotics, and oral beta-lactamases or other antibiotic-degrading enzymes [126–128]. PT utilizes bacteriophages that selectively target pathogenic bacteria without harming commensal bacteria [126]. Bacteriophages are natural predators of bacteria that have coevolved with bacteria for billions of years [129,130]. Phages were used to treat infections like bacterial dysentery in 1919, long before the discovery of penicillin in 1928 [130]. Phages disrupt bacterial biofilm formation and render bacteria susceptible to antibiotics, thereby facilitating their clearance [130]. Due to an increase in antimicrobial resistance (AMR), studies on phage therapy have been growing substantially. Since 2020, around 29 clinical trials have been registered investigating the use of phages as bactericidal agents [130]. However, PT has some limitations, such as a narrow host spectrum, easy clearance of bacteriophage by the immune system, and evolution of anti-phage bacterial strains [126]. Additionally, bacteria develop resistance to bacteriophages through modulating phage receptors via receptor mutations or bacterial clustered regularly interspaced short palindromic repeats (CRISPR) – CRISPR-associated (Cas) system [131]. Moreover, phages require specific conditions for their stability and lytic activity, such as a pH of 6–8 and a certain range of temperature [126,132]. In addition, the use of PT as a standard therapeutic option is not currently available due to safety concerns and absence of appropriate legal and regulatory framework [130].

Moreover, narrow-spectrum antibiotics can minimize dysbiosis by targeting harmful bacteria while sparing commensal bacteria. For instance, narrow-spectrum antibiotics like Fidaxomicin are used for treating *Clostridioides difficile* infection instead of broad-spectrum antibiotics [127]. In addition, to prevent antibiotic-induced dysbiosis, oral beta-lactamases can be used to degrade remaining antibiotics in the gut during treatment [128].

### Role of prebiotics, probiotics, synbiotics, and postbiotics in enhancing commensals and suppressing pathogens

Prebiotics are non-digestible food ingredients that stimulate the growth and activity of commensal bacteria [133]. Several examples of foods can be classified as prebiotics include inulin and fructo-oligosaccharides [133]. To qualify as a prebiotic, a food substance must be able to tolerate gastric acidity and resist hydrolysis by mammalian enzymes [133]. Prebiotics must also be fermented by intestinal microbiota and selectively promote the growth of beneficial bacteria [133]. They contribute to the production of SCFAs such as butyrate, acetate, and propionate, which have significant effects on the host’s health. SCFAs support the integrity of the gut barrier and mucus layer [134,135]. Additionally, SCFAs play an essential role in regulating immune responses and maintaining homeostasis. They reduce ROS production by neutrophils and increase the generation of Tregs [134]. SCFAs also lower gut inflammation by increasing anti-inflammatory cytokines like IL-10 [134]. Importantly, SCFAs lower the pH, which promotes the growth of *Bifidobacteria* and *Lactobacilli* while inhibiting pathogenic bacteria [83,136–138]. By enhancing the growth of beneficial bacteria, prebiotics increase the population of commensals.

Probiotics are nonpathogenic microbes that confer health benefits when administered in adequate amounts [139]. Various kinds of food contain probiotics, such as yogurt, kefir, fermented pickles, and cheese. Probiotics, such as *Lactobacillus* and *Bifidobacterium*, enhance commensals in various ways. They compete for binding sites on the mucosal layer, thereby offering native commensals an opportunity to grow and maintain intestinal homeostasis [140]. Probiotics indirectly kill pathogenic bacteria like *Listeria, Clostridium*, and *Salmonella* by producing antimicrobial peptides and bacteriocins [91]. Additionally, probiotics restore the acidic environment in the stomach, which prevents the growth of many pathogenic bacteria [141]. Moreover, probiotics restore diversity and microbial balance after antibiotic-induced dysbiosis [142].

Synbiotics are a combination of prebiotics and probiotics used to restore a healthy microbiome in the gut [143]. The use of synbiotics ensures the survival of probiotics in the gastrointestinal tract [144]. Synbiotics enhance the persistence, diversity, and stability of commensals like *Bifidobacterium* or *Lactobacillus* in the gut [144]. By diversifying the microbiome, synbiotics contribute to a healthy gut, especially after antibiotic-induced dysbiosis. Synbiotics selectively increase the population of diverse commensals, which prevent colonization and invasion by pathogenic bacteria. In a pre-clinical study, it was shown that supplementation of synbiotics to rats with antibiotic-associated gut dysbiosis significantly improved gut health [145].

Postbiotics are microbial byproducts and bioactive compounds released by probiotics [146]. Postbiotics include several non-viable constituents such as SCFAs, metabolites, functional proteins, extracellular polysaccharides (EPS), enzymes, peptides, and vitamins [146–148]. They play a crucial role in enhancing the functions of commensals, such as regulating metabolism, enhancing immunological functions, promoting antioxidant effects, and exerting anticancer effects in various cancers, including gastric, hematologic, breast, and colon cancer [146,147,149]. Postbiotics can also induce immunomodulatory effects by inducing anti-inflammatory responses [147]. Through the release of antimicrobial peptides, organic acids, bacteriocins, hydrogen peroxide, and fatty acids, postbiotics suppress the growth of important pathogenic bacteria, like *E. coli* and *S. enteritidis* [150,151]. Postbiotics could also be used to alleviate dysbiosis caused by antibiotic use, poor diet, or infections [152].

### Commensals as opportunistic pathogens

Although the presence of commensal bacteria is essential for host immunity, especially in the gut, their disruption can lead to opportunistic infections. Opportunistic infections occur mostly in immunocompromised individuals, such as those with human immunodeficiency virus (HIV), organ transplantation, wounds, burns, diabetes, cancer, and surgery. Additionally, one of the most common triggers for opportunistic infections is the use of antibiotics. UTIs and abdominal infections are caused by several opportunistic bacteria, including *E. coli*, *Enterococcus faecalis*, and *Bacteroides fragilis* [153,154]. *Cutibacterium acnes* is also an opportunistic pathogen that cause acne or endocarditis [155]. In addition, *Staphylococcus epidermidis* is considered harmless; however, under certain circumstances, such as surgery, it can cause infections related to medical devices (i.e. catheter) [156,157].

## Concluding remarks

The immune system plays a crucial role in defending the host against invading bacteria while maintaining immune tolerance toward beneficial bacteria. The human body continually interacts with microbes, especially bacteria. The type of bacteria influences subsequent immunomodulatory events, which can result in protective or regulatory immune responses. For instance, pathogenic bacteria like *salmonella* and *shigella* trigger pro-inflammatory responses through the NF-κB signaling pathway, whereas beneficial bacteria such as *Lactobacillus* spp. and *Bifidobacterium* spp. promote regulatory and anti-inflammatory immune responses [158–160]. The initial microbial engagement with the host starts by recognizing and binding of bacterial components to host cell receptors such as TLR4. This recognition promotes cells, including epithelial cells, to send warning signals (i.e. cytokines) to dendritic cells, T cells, and B cells, ultimately shaping adaptive immune responses. Bacteria have specific features that facilitate immune discrimination. Commensals have characteristics that protect them from being destroyed by the immune system, including their location within the body (e.g. being confined to the gut lumen), the lack of virulence factors and toxins, and their inability to breach host tissues and cause harm. Commensals protect the host from invading pathogens via various mechanisms, including competing for space and nutrients, producing antimicrobial peptides, maintaining intestinal homeostasis, and contributing to immune cell development. Disruption of commensals in the intestine causes dysbiosis. Antibiotic use is the most common cause of dysbiosis. Administration of biotics such as prebiotics, probiotics, synbiotics, and postbiotics can help treat dysbiosis by restoring microbial balance and intestinal homeostasis.

## Data Availability

Data sharing is not applicable to this article as no new data were created or analyzed in this study.

## References

[1] Silva MT, Correia-Neves M. Neutrophils and macrophages: the main partners of phagocyte cell systems. Front Immunol. 2012;3:174. doi: 10.3389/fimmu.2012.00174 22783254 PMC3389340

[2] Nguyen GT, Green ER, Mecsas J. Neutrophils to the ROScue: mechanisms of NADPH oxidase activation and bacterial resistance. Front Cell Infect Microbiol. 2017;7:373. doi: 10.3389/fcimb.2017.00373 28890882 PMC5574878

[3] Du C, Lu M, Zheng J, et al. Distinctive features and prognostic utility of neutrophil in severe patients with Klebsiella pneumoniae infection. Front Cell Infect Microbiol. 2024;14:Volume14–15. doi: 10.3389/fcimb.2024.1406168 PMC11405363 39290978

[4] Wicherska-Pawłowska K, Wróbel T, Rybka J. Toll-like receptors (TLRs), NOD-like receptors (NLRs), and RIG-I-like receptors (RLRs) in innate immunity. TLRs, NLRs, and RLRs ligands as immunotherapeutic agents for hematopoietic diseases. Int J Mol Sci. 2021;22(24):13397. doi: 10.3390/ijms222413397 34948194 PMC8704656

[5] Li D, Wu M. Pattern recognition receptors in health and diseases. Signal Transduct Target Ther. 2021;6(1):291. doi: 10.1038/s41392-021-00687-0 PMC8333067 34344870

[6] Fu YL, Harrison RE. Microbial phagocytic receptors and their potential involvement in cytokine induction in macrophages. Front Immunol. 2021;12:662063. doi: 10.3389/fimmu.2021.662063 33995386 PMC8117099

[7] Markiewski MM, Lambris JD. The role of complement in inflammatory diseases from behind the scenes into the spotlight. Am J Pathol. 2007;171(3):715–727. doi: 10.2353/ajpath.2007.070166 17640961 PMC1959484

[8] Netea MG, Schlitzer A, Placek K, et al. Innate and adaptive immune memory: an evolutionary continuum in the host’s response to pathogens. Cell Host Microbe. 2019;25(1):13–26. doi: 10.1016/j.chom.2018.12.006 30629914

[9] Itano AA, McSorley SJ, Reinhardt RL, et al. Distinct dendritic cell populations sequentially present antigen to CD4 T cells and stimulate different aspects of cell-mediated immunity. Immunity. 2003;19(1):47–57. doi: 10.1016/S1074-7613(03)00175-4 12871638

[10] Waibl Polania J, Hoyt-Miggelbrink A, Tomaszewski WH, et al. Antigen presentation by tumor-associated macrophages drives T cells from a progenitor exhaustion state to terminal exhaustion. Immunity. 2025;58(1):232–246.e6. doi: 10.1016/j.immuni.2024.11.026 39724910

[11] Tippalagama R, Chihab LY, Kearns K, et al. Antigen-specificity measurements are the key to understanding T cell responses. Front Immunol. 2023;14:Volume14–2023. doi: 10.3389/fimmu.2023.1127470 PMC10140422 37122719

[12] Pavlova AV, Zvyagin IV, Shugay M. Detecting T-cell clonal expansions and quantifying clone survival using deep profiling of immune repertoires. Front Immunol. 2024;15:1321603. doi: 10.3389/fimmu.2024.1321603 38633256 PMC11021634

[13] Ferapontov A, Omer M, Baudrexel I, et al. Antigen footprint governs activation of the B cell receptor. Nat Commun. 2023;14(1):976. doi: 10.1038/s41467-023-36672-0 36813795 PMC9947222

[14] Akkaya M, Kwak K, Pierce SK. B cell memory: building two walls of protection against pathogens. Nat Rev Immunol. 2020;20(4):229–238. doi: 10.1038/s41577-019-0244-2 31836872 PMC7223087

[15] Wade WF, O’Toole GA. Antibodies and immune effectors: shaping Gram-negative bacterial phenotypes. Trends Microbiol. 2010;18(6):234–239. doi: 10.1016/j.tim.2010.03.001 20359893 PMC8962613

[16] Forthal DN. Functions of antibodies. Microbiol Spectr. 2014;2(4):1–17. doi: 10.1128/microbiolspec.AID-0019-2014 PMC4159104 25215264

[17] Kaech SM, Wherry EJ, Ahmed R. Effector and memory T-cell differentiation: implications for vaccine development. Nat Rev Immunol. 2002;2(4):251–262. doi: 10.1038/nri778 12001996

[18] Khan R, Petersen FC, Shekhar S. Commensal bacteria: an emerging player in defense against respiratory pathogens. Front Immunol. 2019;10:1203. doi: 10.3389/fimmu.2019.01203 31214175 PMC6554327

[19] Hou K, Wu Z-X, Chen X-Y, et al. Microbiota in health and diseases. Signal Transduct Target Ther. 2022;7(1):135. doi: 10.1038/s41392-022-00974-4 35461318 PMC9034083

[20] Fung TC, Artis D, Sonnenberg GF. Anatomical localization of commensal bacteria in immune cell homeostasis and disease. Immunol Rev. 2014;260(1):35–49. doi: 10.1111/imr.12186 24942680 PMC4216679

[21] Chen Y, Cui W, Li X, et al. Interaction between commensal bacteria, immune response and the intestinal barrier in inflammatory bowel disease. Front Immunol. 2021;12:761981. doi: 10.3389/fimmu.2021.761981 34858414 PMC8632219

[22] Popoff MR. Overview of bacterial protein toxins from pathogenic bacteria: mode of action and insights into evolution. Toxins (Basel). 2024;16(4):182. doi: 10.3390/toxins16040182 38668607 PMC11054074

[23] Anjana A, Tiwari SK. Bacteriocin-producing probiotic lactic acid bacteria in controlling dysbiosis of the gut microbiota. Front Cell Infect Microbiol. 2022;12:851140. doi: 10.3389/fcimb.2022.851140 35651753 PMC9149203

[24] Zhang Y, He Z. Inflammatory mediators in bacterial vaginosis: the role of cytokines. APMIS. 2024;132(4):245–255. doi: 10.1111/apm.13380 38345182

[25] Pan M, Barua N, Ip M. Mucin-degrading gut commensals isolated from healthy faecal donor suppress intestinal epithelial inflammation and regulate tight junction barrier function. Front Immunol. 2022;13:1021094. doi: 10.3389/fimmu.2022.1021094 36311778 PMC9597641

[26] Ness S, Lin S, Gordon JR. Regulatory dendritic cells, T cell tolerance, and dendritic cell therapy for immunologic disease. Front Immunol. 2021;12:633436. doi: 10.3389/fimmu.2021.633436 33777019 PMC7988082

[27] Mbongue JC, Vanterpool E, Firek A, et al. Lipopolysaccharide-induced immunological tolerance in monocyte-derived dendritic cells. Immuno. 2022;2(3):482–500. doi: 10.3390/immuno2030030

[28] Luciani C, Hager FT, Cerovic V, et al. Dendritic cell functions in the inductive and effector sites of intestinal immunity. Mucosal Immunol. 2022;15(1):40–50. doi: 10.1038/s41385-021-00448-w 34465895

[29] Liu J, Zhang X, Cheng Y, et al. Dendritic cell migration in inflammation and immunity. Cell Mol Immunol. 2021;18(11):2461–2471. doi: 10.1038/s41423-021-00726-4 34302064 PMC8298985

[30] Takeuchi T, Ohno H. IgA in human health and diseases: potential regulator of commensal microbiota. Front Immunol. 2022;13:1024330. doi: 10.3389/fimmu.2022.1024330 36439192 PMC9685418

[31] Hu J, Ye Y, Chen X, et al. Insight into the pathogenic mechanism of Mycoplasma pneumoniae. Curr Microbiol. 2023;80(1):14. doi: 10.1007/s00284-022-03103-0 PMC9716528 36459213

[32] Rautava S, Walker WA. Commensal bacteria and epithelial cross talk in the developing intestine. Curr Gastroenterol Rep. 2007;9(5):385–392. doi: 10.1007/s11894-007-0047-7 17991339 PMC3208513

[33] Zheng D, Liwinski T, Elinav E. Interaction between microbiota and immunity in health and disease. Cell Res. 2020;30(6):492–506. doi: 10.1038/s41422-020-0332-7 32433595 PMC7264227

[34] Arpaia N, Campbell C, Fan X, et al. Metabolites produced by commensal bacteria promote peripheral regulatory T-cell generation. Nature. 2013;504(7480):451–455. doi: 10.1038/nature12726 24226773 PMC3869884

[35] Yuk J-M, Yoshimori T, Jo E-K. Autophagy and bacterial infectious diseases. Exp Mol Med. 2012;44(2):99–108. doi: 10.3858/emm.2012.44.2.032 22257885 PMC3296818

[36] Vasquez Ayala A, Hsu C-Y, Oles RE, et al. Commensal bacteria promote type I interferon signaling to maintain immune tolerance in mice. J Exp Med. 2023;221(1):e20230063. doi: 10.1084/jem.20230063 38085267 PMC10716256

[37] Shim JA, Ryu JH, Jo Y, et al. The role of gut microbiota in T cell immunity and immune mediated disorders. Int J Biol Sci. 2023;19(4):1178. doi: 10.7150/ijbs.79430 36923929 PMC10008692

[38] Zhao Q, Elson CO. Adaptive immune education by gut microbiota antigens. Immunology. 2018;154(1):28–37. doi: 10.1111/imm.12896 29338074 PMC5904715

[39] Hand TW, Reboldi A. Production and function of immunoglobulin A. Annu Rev Immunol. 2021;39(1):695–718. doi: 10.1146/annurev-immunol-102119-074236 33646857

[40] Ohno H. Intestinal M cells. J Biochem. 2016;159(2):151–160. doi: 10.1093/jb/mvv121 26634447 PMC4892784

[41] Gieryńska M, Szulc-Dąbrowska L, Struzik J, et al. Integrity of the intestinal barrier: the involvement of epithelial cells and microbiota—a mutual relationship. Animals. 2022;12(2):145. doi: 10.3390/ani12020145 35049768 PMC8772550

[42] Tonetti FR, Eguileor A, Llorente C. Goblet cells: guardians of gut immunity and their role in gastrointestinal diseases. Egastroenterology. 2024;2(3):e100098. doi: 10.1136/egastro-2024-100098 39524932 PMC11542612

[43] Coutry N, Gasmi I, Herbert F, et al. Mechanisms of intestinal dysbiosis: new insights into tuft cell functions. Gut Microbes. 2024;16(1):2379624. doi: 10.1080/19490976.2024.2379624 39042424 PMC11268228

[44] Wiertsema SP, van Bergenhenegouwen J, Garssen J, et al. The interplay between the gut microbiome and the immune system in the context of infectious diseases throughout life and the role of nutrition in optimizing treatment strategies. Nutrients. 2021;13(3):886. doi: 10.3390/nu13030886 33803407 PMC8001875

[45] Granucci F, Zanoni I, Ricciardi-Castagnoli P. Central role of dendritic cells in the regulation and deregulation of immune responses. Cell Mol Life Sci. 2008;65(11):1683–1697. doi: 10.1007/s00018-008-8009-2 18327662 PMC11131678

[46] Abreu MT. Toll-like receptor signalling in the intestinal epithelium: how bacterial recognition shapes intestinal function. Nat Rev Immunol. 2010;10(2):131–144. doi: 10.1038/nri2707 20098461

[47] Sameer AS, Nissar S. Toll-like receptors (TLRs): structure, functions, signaling, and role of their polymorphisms in colorectal cancer susceptibility. Biomed Res Int. 2021;2021(1):1157023. doi: 10.1155/2021/1157023 34552981 PMC8452412

[48] Le Noci V, Bernardo G, Bianchi F, et al. Toll like receptors as sensors of the tumor microbial dysbiosis: implications in cancer progression. Front Cell Dev Biol. 2021;9:732192. doi: 10.3389/fcell.2021.732192 34604233 PMC8485072

[49] Rakoff-Nahoum S, Paglino J, Eslami-Varzaneh F, et al. Recognition of commensal microflora by toll-like receptors is required for intestinal homeostasis. Cell. 2004;118(2):229–241. doi: 10.1016/j.cell.2004.07.002 15260992

[50] Chen X, Berin MC, Gillespie VL, et al. Treatment of intestinal inflammation with epicutaneous immunotherapy requires TGF-β and IL-10 but not Foxp3+ Tregs. Front Immunol. 2021;12:637630. doi: 10.3389/fimmu.2021.637630 33717186 PMC7952322

[51] Lee YM, Almqvist F, Hultgren SJ. Targeting virulence for antimicrobial chemotherapy. Curr Opin Pharmacol. 2003;3(5):513–519. 14559097 10.1016/j.coph.2003.04.001

[52] Vasquez Ayala A, Hsu C-Y, Oles RE, et al. Commensal bacteria promote type I interferon signaling to maintain immune tolerance in mice. J Exp Med. 2023;221(1). doi: 10.1084/jem.20230063 PMC10716256 38085267

[53] Chen L, Zhang L, Hua H, et al. Interactions between toll-like receptors signaling pathway and gut microbiota in host homeostasis. Immun Inflamm Dis. 2024;12(7):e1356. doi: 10.1002/iid3.1356 39073297 PMC11284964

[54] Zheng D, Kern L, Elinav E. The NLRP6 inflammasome. Immunology. 2021;162(3):281–289. doi: 10.1111/imm.13293 33314083 PMC7884648

[55] Krishnamurthy HK, Pereira M, Bosco J, et al. Gut commensals and their metabolites in health and disease. Front Microbiol. 2023;14:1244293. doi: 10.3389/fmicb.2023.1244293 38029089 PMC10666787

[56] Flint HJ, Scott KP, Louis P, et al. The role of the gut microbiota in nutrition and health. Nat Rev Gastroenterol Hepatol. 2012;9(10):577–589. doi: 10.1038/nrgastro.2012.156 22945443

[57] Jandhyala SM. Role of the normal gut microbiota. World J Gastroenterol. 2015;21(29):8787–8803. doi: 10.3748/wjg.v21.i29.8787 26269668 PMC4528021

[58] Galleher C. Gut microbiome and its role in enteric infections with microbial pathogens. In: Gut microbiome and its impact on health and diseases. 2020. p. 187–208.

[59] Closs G, Bhandari M, Helmy YA, et al. The probiotic *Lacticaseibacillus rhamnosus* GG supplementation reduces *Salmonella* load and modulates growth, intestinal morphology, gut microbiota, and immune responses in chickens. Infect Immun. 2025;93(5):e00420–24. doi: 10.1128/iai.00420-24 40172512 PMC12070740

[60] Shi S, Gong L, Yu H, et al. Antagonistic activity and mechanism of Lactobacillus rhamnosus SQ511 against Salmonella enteritidis. 3 Biotech. 2022;12(6):126. doi: 10.1007/s13205-022-03176-5 PMC9095802 35573802

[61] Liu D, Li C, Cao T, et al. Bifidobacterium longum K5 prevents enterohaemorrhagic Escherichia coli O157: h7 infection in mice through the modulation of the gut microbiota. Nutrients. 2024;16(8):1164. doi: 10.3390/nu16081164 38674854 PMC11053520

[62] Rouhi A, Falah F, Azghandi M, et al. Investigating the effect of Lactiplantibacillus plantarum TW57-4 in preventing biofilm formation and expression of virulence genes in Listeria monocytogenes ATCC 19115. LWT. 2024;191:115669. doi: 10.1016/j.lwt.2023.115669

[63] Horn KJ, Jaberi Vivar AC, Arenas V, et al. Corynebacterium species inhibit Streptococcus pneumoniae colonization and infection of the mouse airway. Front Microbiol. 2022;12:Volume12–2021. doi: 10.3389/fmicb.2021.804935 PMC8784410 35082772

[64] Huang S, Hon K, Bennett C, et al. Corynebacterium accolens inhibits Staphylococcus aureus induced mucosal barrier disruption. Front Microbiol. 2022;13:Volume13–2022. doi: 10.3389/fmicb.2022.984741 PMC9515799 36187946

[65] Saenz C, Fang Q, Gnanasekaran T, et al. *Clostridium scindens* secretome suppresses virulence gene expression of *Clostridioides difficile* in a bile acid-independent manner. Microbiol Spectr. 2023;11(5):e03933–22. doi: 10.1128/spectrum.03933-22 37750706 PMC10581174

[66] Johnson JA, Delaney LF, Ojha V, et al. Commensal urinary lactobacilli inhibit major uropathogens in vitro with heterogeneity at species and strain level. Front Cell Infect Microbiol. 2022;12:Volume12–2022. doi: 10.3389/fcimb.2022.870603 PMC9260849 35811675

[67] Luo S-C, Wei S-M, Luo X-T, et al. How probiotics, prebiotics, synbiotics, and postbiotics prevent dental caries: an oral microbiota perspective. NPJ Biofilms Microbiomes. 2024;10(1):14. doi: 10.1038/s41522-024-00488-7 38402294 PMC10894247

[68] Zipperer A, Konnerth MC, Laux C, et al. Human commensals producing a novel antibiotic impair pathogen colonization. Nature. 2016;535(7613):511–516. doi: 10.1038/nature18634 27466123

[69] Ubeda C, Djukovic A, Isaac S. Roles of the intestinal microbiota in pathogen protection. Clin Transl Immunol. 2017;6(2):e128. doi: 10.1038/cti.2017.2 PMC5311919 28243438

[70] Kommineni S, Bretl DJ, Lam V, et al. Bacteriocin production augments niche competition by enterococci in the mammalian gastrointestinal tract. Nature. 2015;526(7575):719–722. doi: 10.1038/nature15524 26479034 PMC4978352

[71] Zhang H, Pan Y, Jiang Y, et al. Akkermansia muciniphila one effectively ameliorates dextran sulfate sodium (DSS)-induced ulcerative colitis in mice. Npj Sci Food. 2024;8(1):97. doi: 10.1038/s41538-024-00339-x 39562574 PMC11576909

[72] Zhang W, Zhou Q, Liu H, et al. Bacteroides fragilis strain ZY-312 facilitates colonic mucosa regeneration in colitis via motivating STAT3 signaling pathway induced by IL-22 from ILC3 secretion. Front Immunol. 2023;14:1156762. doi: 10.3389/fimmu.2023.1156762 37114045 PMC10126674

[73] Dempsey E, Corr SC. Lactobacillus spp. for gastrointestinal health: current and future perspectives. Front Immunol. 2022;13:840245. doi: 10.3389/fimmu.2022.840245 35464397 PMC9019120

[74] Lopez-Siles M, Duncan SH, Garcia-Gil LJ, et al. Faecalibacterium prausnitzii: from microbiology to diagnostics and prognostics. Isme J. 2017;11(4):841–852. doi: 10.1038/ismej.2016.176 28045459 PMC5364359

[75] Mo C, Lou X, Xue J, et al. The influence of Akkermansia muciniphila on intestinal barrier function. Gut Pathog. 2024;16(1):41. doi: 10.1186/s13099-024-00635-7 39097746 PMC11297771

[76] Breugelmans T, Oosterlinck B, Arras W, et al. The role of mucins in gastrointestinal barrier function during health and disease. Lancet Gastroenterol Hepatol. 2022;7(5):455–471. doi: 10.1016/S2468-1253(21)00431-3 35397245

[77] Prame Kumar K, Ooi JD, Goldberg R. The interplay between the microbiota, diet and T regulatory cells in the preservation of the gut barrier in inflammatory bowel disease. Front Microbiol. 2023;14:1291724. doi: 10.3389/fmicb.2023.1291724 38107848 PMC10722198

[78] Nakajima A, Sasaki T, Itoh K, et al. A soluble fiber diet increases Bacteroides fragilis group abundance and immunoglobulin A production in the gut. Appl Environ Microbiol. 2020;86(13). doi: 10.1128/AEM.00405-20 PMC7301863 32332136

[79] Ding M, Li B, Chen H, et al. Bifidobacterium longum subsp. infantis promotes IgA level of growing mice in a strain-specific and intestinal niche-dependent manner. Nutrients. 2024;16(8):1148. doi: 10.3390/nu16081148 38674840 PMC11054607

[80] Mei L, Chen Y, Wang J, et al. Lactobacillus fermentum stimulates intestinal secretion of immunoglobulin A in an individual-specific manner. Foods. 2022;11(9):1229. doi: 10.3390/foods11091229 35563952 PMC9099657

[81] Canales-Herrerias P, Cerutti A. Gut IgA: never fear, the super inducers are here. Cell Host Microbe. 2023;31(10):1595–1597. doi: 10.1016/j.chom.2023.09.004 37827118

[82] Lee B-H, Hu Y-F, Chu Y-T, et al. Lactic acid bacteria-fermented diet containing bacterial extracellular vesicles inhibited pathogenic bacteria in striped beakfish (Oplegnathus fasciatus). Fermentation. 2024;10(1):49. doi: 10.3390/fermentation10010049

[83] Deleu S, Machiels K, Raes J, et al. Short chain fatty acids and its producing organisms: an overlooked therapy for IBD? EBioMedicine. 2021;66:103293. doi: 10.1016/j.ebiom.2021.103293 33813134 PMC8047503

[84] Tufail MA, Schmitz RA. Exploring the probiotic potential of Bacteroides spp. One Health Para Probiotics Antimicrob Proteins. 2024;17(2):681–704. doi: 10.1007/s12602-024-10370-9 PMC11925995 39377977

[85] Zafar H, Saier MH. Gut Bacteroides species in health and disease. Gut Microbes. 2021;13(1):1–20. doi: 10.1080/19490976.2020.1848158 PMC7872030 33535896

[86] Guo L, Liu Q, Yin X. Gut microbiota protects Listeria monocytogenes-infected mice by reducing the inflammatory cytokines storm and cell apoptosis. Foodborne Pathog Dis. 2024;21(5):288–297. doi: 10.1089/fpd.2023.0121 38237167

[87] Horrocks V, King OG, Yip AYG, et al. Role of the gut microbiota in nutrient competition and protection against intestinal pathogen colonization. Microbiol (Read). 2023;169(8). doi: 10.1099/mic.0.001377 PMC10482380 37540126

[88] Horrocks V, King OG, Yip AYG, et al. Role of the gut microbiota in nutrient competition and protection against intestinal pathogen colonization. Microbiology. 2023;169(8):001377. doi: 10.1099/mic.0.001377 37540126 PMC10482380

[89] Ogawa M, Shimizu K, Nomoto K, et al. Inhibition of in vitro growth of shiga toxin-producing Escherichia coli O157: h7 by probiotic Lactobacillus strains due to production of lactic acid. Int J Food Microbiol. 2001;68(1–2):135–140. doi: 10.1016/S0168-1605(01)00465-2 11545213

[90] Brownlie EJE, Chaharlangi D, Wong EO-Y, et al. Acids produced by lactobacilli inhibit the growth of commensal lachnospiraceae and S24-7 bacteria. Gut Microbes. 2022;14(1):2046452. doi: 10.1080/19490976.2022.2046452 35266847 PMC8920129

[91] Anjana, Tiwari SK. Bacteriocin-producing probiotic lactic acid bacteria in controlling dysbiosis of the gut microbiota. Front Cell Infect Microbiol. 2022;12:851140. 35651753 10.3389/fcimb.2022.851140 PMC9149203

[92] Khan F, Singh P, Joshi AS, et al. Multiple potential strategies for the application of nisin and derivatives. Crit Rev Microbiol. 2023;49(5):628–657. doi: 10.1080/1040841X.2022.2112650 35997756

[93] Huang F, Teng K, Liu Y, et al. Bacteriocins: potential for human health. Oxid Med Cell Longev. 2021;2021(1):5518825. doi: 10.1155/2021/5518825 33936381 PMC8055394

[94] Parker JK, Feller AL, Gu R, et al. Antibacterial microcins are ubiquitous and functionally diverse across bacterial communities. Nat Commun. 2025;16(1):6048. doi: 10.1038/s41467-025-61151-z 40595659 PMC12219008

[95] Miko E, Barakonyi A. The role of hydrogen-peroxide (H2O2) produced by vaginal microbiota in female reproductive health. Antioxidants. 2023;12(5):1055. doi: 10.3390/antiox12051055 37237921 PMC10215881

[96] Borreby C, Lillebæk EMS, Kallipolitis BH. Anti-infective activities of long-chain fatty acids against foodborne pathogens. FEMS Microbiol Rev. 2023;47(4):fuad037. doi: 10.1093/femsre/fuad037 37437907 PMC10368373

[97] Zhao Y, Bitzer A, Power JJ, et al. Nasal commensals reduce Staphylococcus aureus proliferation by restricting siderophore availability. Isme J. 2024;18(1). doi: 10.1093/ismejo/wrae123 PMC11296517 38987933

[98] Anumudu CK, Miri T, Onyeaka H. Multifunctional applications of lactic acid bacteria: enhancing safety, quality, and nutritional value in foods and fermented beverages. Foods. 2024;13(23):3714. doi: 10.3390/foods13233714 39682785 PMC11640447

[99] Bidell MR, Hobbs AL, Lodise TP. Gut microbiome health and dysbiosis: a clinical primer. Pharmacother J Hum Pharmacol Drug Ther. 2022;42(11):849–857. doi: 10.1002/phar.2731 PMC9827978 36168753

[100] Lin X, Lin C, Li X, et al. Gut microbiota dysbiosis facilitates susceptibility to bloodstream infection. J Microbiol. 2024;62(12):1113–1124. doi: 10.1007/s12275-024-00190-5 39621250 PMC11652583

[101] Zhang F, Lau RI, Liu Q, et al. Gut microbiota in COVID-19: key microbial changes, potential mechanisms and clinical applications. Nat Rev Gastroenterol Hepatol. 2023;20(5):323–337. doi: 10.1038/s41575-022-00698-4 36271144 PMC9589856

[102] Li R, Li J, Zhou X. Lung microbiome: new insights into the pathogenesis of respiratory diseases. Signal Transduct Target Ther. 2024;9(1):19. doi: 10.1038/s41392-023-01722-y 38228603 PMC10791971

[103] Loh JS, Mak WQ, Tan LKS, et al. Microbiota–gut–brain axis and its therapeutic applications in neurodegenerative diseases. Signal Transduct Target Ther. 2024;9(1):37. doi: 10.1038/s41392-024-01743-1 38360862 PMC10869798

[104] Huang Y, Liao J, Liu X, et al. Review: the role of intestinal dysbiosis in Parkinson’s disease. Front Cell Infect Microbiol. 2021;11:615075. doi: 10.3389/fcimb.2021.615075 33968794 PMC8100321

[105] Carding S, Verbeke K, Vipond DT, et al. Dysbiosis of the gut microbiota in disease. Microb Ecol Health Dis. 2015;26(1):26191. doi: 10.3402/mehd.v26.26191 25651997 PMC4315779

[106] DeGruttola AK, Low D, Mizoguchi A, et al. *Current understanding of dysbiosis in disease in human and animal models*. Inflammatory bowel diseases. Inflamm Bowel Dis. 2016;22(5):1137–1150. doi: 10.1097/MIB.0000000000000750 27070911 PMC4838534

[107] Ohland CL, MacNaughton WK. Probiotic bacteria and intestinal epithelial barrier function. Am J Physiol-Gastrointestinal Liver Physiol. 2010;298(6):G807–G819. doi: 10.1152/ajpgi.00243.2009 20299599

[108] Fayol-Messaoudi D, Berger CN, Coconnier-Polter M-H, et al. pH-, lactic acid-, and non-lactic acid-dependent activities of probiotic lactobacilli against Salmonella enterica serovar Typhimurium. Appl Environ Microbiol. 2005;71(10):6008–6013. doi: 10.1128/AEM.71.10.6008-6013.2005 16204515 PMC1266002

[109] Lievin V, Peiffer I, Hudault S, et al. Bifidobacterium strains from resident infant human gastrointestinal microflora exert antimicrobial activity. Gut. 2000;47(5):646–652. doi: 10.1136/gut.47.5.646 11034580 PMC1728100

[110] Mann ER, Li X. Intestinal antigen-presenting cells in mucosal immune homeostasis: crosstalk between dendritic cells, macrophages and B-cells. World J Gastroenterol: wJG. 2014;20(29):9653. doi: 10.3748/wjg.v20.i29.9653 25110405 PMC4123356

[111] Chang S-H, Choi Y. Gut dysbiosis in autoimmune diseases: association with mortality. Front Cell Infect Microbiol. 2023;13:1157918. doi: 10.3389/fcimb.2023.1157918 37065187 PMC10102475

[112] Mousa WK, Chehadeh F, Husband S. Microbial dysbiosis in the gut drives systemic autoimmune diseases. Front Immunol. 2022;13:906258. doi: 10.3389/fimmu.2022.906258 36341463 PMC9632986

[113] de Oliveira GLV, Leite AZ, Higuchi BS, et al. Intestinal dysbiosis and probiotic applications in autoimmune diseases. Immunology. 2017;152(1):1–12. doi: 10.1111/imm.12765 28556916 PMC5543467

[114] Kaur S, Kumari D, Dandekar MP. Importance of gut microbiota dysbiosis and circadian disruption–associated biomarkers in emergence of Alzheimer’s disease. Mol Neurobiol. 2025;62(5):1–9. doi: 10.1007/s12035-024-04685-5 39775480

[115] Bien J, Palagani V, Bozko P. The intestinal microbiota dysbiosis and Clostridium difficile infection: is there a relationship with inflammatory bowel disease? Therap Adv Gastroenterol. 2013;6(1):53–68. doi: 10.1177/1756283X12454590 23320050 PMC3539291

[116] Horta-Baas G, Romero-Figueroa MDS, Montiel-Jarquín AJ, et al. Intestinal dysbiosis and rheumatoid arthritis: a link between gut microbiota and the pathogenesis of rheumatoid arthritis. J Immunol Res. 2017;2017:4835189. doi: 10.1155/2017/4835189 28948174 PMC5602494

[117] Chandrasekhar D, Joseph CM, Parambil JC, et al. Superbugs: an invicible threat in post antibiotic era. Clin Epidemiol Global Health. 2024;28:101499. doi: 10.1016/j.cegh.2023.101499

[118] Cong L, Chen C, Mao S, et al. Intestinal bacteria-a powerful weapon for fungal infections treatment. Front Cell Infect Microbiol. 2023;13:1187831. doi: 10.3389/fcimb.2023.1187831 37333850 PMC10272564

[119] Acevedo-Román A, Pagán-Zayas N, Velázquez-Rivera LI, et al. Insights into gut dysbiosis: inflammatory diseases, obesity, and restoration approaches. Int J Mol Sci. 2024;25(17):9715. doi: 10.3390/ijms25179715 39273662 PMC11396321

[120] Tian H, Wang X, Fang Z, et al. Fecal microbiota transplantation in clinical practice: present controversies and future prospects. hLife. 2024;2(6):269–283. doi: 10.1016/j.hlife.2024.01.006

[121] Song JH, Kim YS. Recurrent Clostridium difficile infection: risk factors, treatment, and prevention. Gut Liver. 2019;13(1):16–24. doi: 10.5009/gnl18071 30400734 PMC6346998

[122] Minkoff NZ, Aslam S, Medina M, et al. Fecal microbiota transplantation for the treatment of recurrent Clostridioides difficile (Clostridium difficile). Cochrane Database Syst Rev. 2023;2023(4):Cd013871. doi: 10.1002/14651858.CD013871.pub2 PMC10125800 37096495

[123] Jain N, Umar TP, Fahner A-F, et al. Advancing therapeutics for recurrent clostridioides difficile infections: an overview of Vowst’s FDA approval and implications. Gut Microbes. 2023;15(1):2232137. doi: 10.1080/19490976.2023.2232137 37431860 PMC10337487

[124] Kim D-Y, Lee S-Y, Lee J-Y, et al. Gut microbiome therapy: fecal microbiota transplantation vs live biotherapeutic products. Gut Microbes. 2024;16(1):2412376. doi: 10.1080/19490976.2024.2412376 39377231 PMC11469438

[125] Karimi M, Shirsalimi N, Hashempour Z, et al. Safety and efficacy of fecal microbiota transplantation (FMT) as a modern adjuvant therapy in various diseases and disorders: a comprehensive literature review. Front Immunol. 2024;15:2024. doi: 10.3389/fimmu.2024.1439176 PMC11464302 39391303

[126] Lin J, Du F, Long M, et al. Limitations of phage therapy and corresponding optimization strategies: a review. Molecules. 2022;27(6):1857. doi: 10.3390/molecules27061857 35335222 PMC8951143

[127] Louie TJ, Miller MA, Mullane KM, et al. Fidaxomicin versus vancomycin for Clostridium difficile infection. N Engl J Med. 2011;364(5):422–431. doi: 10.1056/NEJMoa0910812 21288078

[128] Kaleko M, Bristol JA, Hubert S, et al. Development of SYN-004, an oral beta-lactamase treatment to protect the gut microbiome from antibiotic-mediated damage and prevent Clostridium difficile infection. Anaerobe. 2016;41:58–67. doi: 10.1016/j.anaerobe.2016.05.015 27262694

[129] Naureen Z. Bacteriophages presence in nature and their role in the natural selection of bacterial populations. Acta Biomed. 2020;91(13–s):e2020024. 10.23750/abm.v91i13-S.10819 PMC8023132 33170167

[130] Sarkodie-Addo P, Osman A-H, Aglomasa BC, et al. Phage therapy in the management of respiratory and pulmonary infections: a systematic review. Ther Adv Infect Dis. 2025;12:20499361241307841. doi: 10.1177/20499361241307841 39866829 PMC11760135

[131] Hasan M, Ahn J. Evolutionary dynamics between phages and bacteria as a possible approach for designing effective phage therapies against antibiotic-resistant bacteria. Antibiot (Basel). 2022;11(7):915. doi: 10.3390/antibiotics11070915 PMC9311878 35884169

[132] Ranveer SA, Dasriya V, Ahmad MF, et al. Positive and negative aspects of bacteriophages and their immense role in the food chain. Npj Sci Food. 2024;8(1):1. doi: 10.1038/s41538-023-00245-8 38172179 PMC10764738

[133] Davani-Davari D, Negahdaripour M, Karimzadeh I, et al. Prebiotics: definition, types, sources, mechanisms, and clinical applications. Foods. 2019;8(3):92. doi: 10.3390/foods8030092 30857316 PMC6463098

[134] Fusco W, Lorenzo MB, Cintoni M, et al. Short-chain fatty-acid-producing bacteria: key components of the human gut microbiota. Nutrients. 2023;15(9):2211. doi: 10.3390/nu15092211 37432351 PMC10180739

[135] Ke A, Parreira VR, Goodridge L, et al. Current and future perspectives on the role of probiotics, prebiotics, and synbiotics in controlling pathogenic Cronobacter spp. in infants. Front Microbiol. 2021;12:2021. doi: 10.3389/fmicb.2021.755083 PMC8567173 34745060

[136] den Besten G, van Eunen K, Groen AK, et al. The role of short-chain fatty acids in the interplay between diet, gut microbiota, and host energy metabolism. J Lipid Res. 2013;54(9):2325–2340. doi: 10.1194/jlr.R036012 23821742 PMC3735932

[137] Liu L, Li Q, Yang Y, et al. Biological function of short-chain fatty acids and its regulation on intestinal health of poultry. Front Vet Sci. 2021;8:2021. doi: 10.3389/fvets.2021.736739 PMC8558227 34733901

[138] Nogacka AM, Cuesta I, Gueimonde M, et al. 2-fucosyllactose metabolism by Bifidobacteria promotes lactobacilli growth in co-culture. Microorganisms. 2023;11(11):2659. doi: 10.3390/microorganisms11112659 38004671 PMC10673426

[139] Latif A, Shehzad A, Niazi S, et al. Probiotics: mechanism of action, health benefits and their application in food industries. Front Microbiol. 2023;14:Volume14–2023. doi: 10.3389/fmicb.2023.1216674 PMC10470842 37664108

[140] Yu YY, Ding L-G, Huang Z-Y, et al. Commensal bacteria-immunity crosstalk shapes mucosal homeostasis in teleost fish. Rev Aquacult. 2021;13(4):2322–2343. doi: 10.1111/raq.12570

[141] Koga Y. Microbiota in the stomach and application of probiotics to gastroduodenal diseases. World J Gastroenterol. 2022;28(47):6702–6715. doi: 10.3748/wjg.v28.i47.6702 36620346 PMC9813937

[142] Lathakumari RH, Vajravelu LK, Satheesan A, et al. Antibiotics and the gut microbiome: understanding the impact on human health. Med Microecol. 2024;20:100106. doi: 10.1016/j.medmic.2024.100106

[143] Parhi P, Liu SQ, Choo WS. Synbiotics: effects of prebiotics on the growth and viability of probiotics in food matrices. Bioact Carb Dietary Fibre. 2024;32:100462. doi: 10.1016/j.bcdf.2024.100462

[144] Markowiak P, Śliżewska K. Effects of probiotics, prebiotics, and synbiotics on human health. Nutrients. 2017;9(9):1021. doi: 10.3390/nu9091021 28914794 PMC5622781

[145] Sarangarajan AV, Jain A, Ferreir JL, et al. The synbiotic solution: evaluating safety and efficacy in antibiotic-associated dysbiosis - a randomized, double-blind, placebo-controlled pre-clinical study with Sprague Dawley rats. Pharmanutrition. 2024;29:100402. doi: 10.1016/j.phanu.2024.100402

[146] Garg A. Postbiotics: bioactive compounds and their effects. In: Mishra N, A Garg S Ashique, editors. Role of gut microbiota and postbiotics for colorectal cancer: advancing therapeutic strategies. Springer Nature Switzerland: Cham; 2025. p 155–176.

[147] Wegh CAM, Geerlings SY, Knol J, et al. Postbiotics and their potential applications in early life nutrition and beyond. Int J Mol Sci. 2019;20(19):4673. doi: 10.3390/ijms20194673 31547172 PMC6801921

[148] Aggarwal S, Sabharwal V, Kaushik P, et al. Postbiotics: from emerging concept to application. Front Sustain Food Syst. 2022;6:2022. doi: 10.3389/fsufs.2022.887642

[149] Balendra V, Rosenfeld R, Amoroso C, et al. Postbiotics as adjuvant therapy in cancer care. Nutrients. 2024;16(15):2400. doi: 10.3390/nu16152400 39125280 PMC11314502

[150] Nataraj BH, Ali SA, Behare PV, et al. Postbiotics-parabiotics: the new horizons in microbial biotherapy and functional foods. Microb Cell Fact. 2020;19(1):168. doi: 10.1186/s12934-020-01426-w 32819443 PMC7441679

[151] Jahedi S, Pashangeh S. Bioactivities of postbiotics in food applications: a review. Iran J Microbiol. 2025;17(3):348–357. doi: 10.18502/ijm.v17i3.18816 40612722 PMC12218878

[152] Pattapulavar V, Ramanujam S, Kini B, et al. Probiotic-derived postbiotics: a perspective on next-generation therapeutics. Front Nutr. 2025;12:2025. doi: 10.3389/fnut.2025.1624539 PMC12311805 40747333

[153] Zhou Y, Zhou Z, Zheng L, et al. Urinary tract infections caused by uropathogenic Escherichia coli: mechanisms of infection and treatment options. Int J Mol Sci. 2023;24(13):10537. doi: 10.3390/ijms241310537 37445714 PMC10341809

[154] Kim B, Kim M, Lee K, et al. Clinical outcomes and molecular characteristics of Bacteroides fragilis infections. Alm. 2024;45(2):223–227. doi: 10.3343/alm.2024.0369 PMC11788700 39478317

[155] Mayslich C, Grange PA, Dupin N. Cutibacterium acnes as an opportunistic pathogen: an update of its virulence-associated factors. Microorganisms. 2021;9(2):303. doi: 10.3390/microorganisms9020303 33540667 PMC7913060

[156] Severn MM, Horswill AR. Staphylococcus epidermidis and its dual lifestyle in skin health and infection. Nat Rev Microbiol. 2023;21(2):97–111. doi: 10.1038/s41579-022-00780-3 36042296 PMC9903335

[157] Burke Ó, Zeden MS, O’Gara JP. The pathogenicity and virulence of the opportunistic pathogen Staphylococcus epidermidis. Virulence. 2024;15(1):2359483. doi: 10.1080/21505594.2024.2359483 38868991 PMC11178275

[158] Jouriani FH, Torkamaneh M, Torfeh M, et al. Native Lactobacillus and Bifidobacterium probiotics modulate autophagy genes and exert anti-inflammatory effect. Sci Rep. 2025;15(1):25006. doi: 10.1038/s41598-025-09596-6 40640283 PMC12246250

[159] Ding YH, Qian L-Y, Pang J, et al. The regulation of immune cells by lactobacilli: a potential therapeutic target for anti-atherosclerosis therapy. Oncotarget. 2017;8(35):59915–59928. doi: 10.18632/oncotarget.18346 28938693 PMC5601789

[160] Gopal A, Chidambaram IS, Devaraj N, et al. Shigella dysenteriae infection activates proinflammatory response through β-catenin/NF-κB signaling pathway. PLOS ONE. 2017;12(4):e0174943. doi: 10.1371/journal.pone.0174943 28430783 PMC5400225

